# T1 and T2 Mapping have a higher diagnostic accuracy for the ischaemic area-at-risk in NSTEMI patients compared with dark blood imaging

**DOI:** 10.1186/1532-429X-16-S1-M4

**Published:** 2014-01-16

**Authors:** Jamie Layland, Samuli M Rauhalammi, David Carrick, Aleksandra Radjenovic, Christie McComb, Colin Berry

**Affiliations:** 1 Cardiology, Golden Jubilee Hospital, Glasgow, UK

## Background

T1 and T2 mapping has shown great promise for the identification of acute myocardial infarction. However, most of this work has been performed in patients with ST-elevation myocardial infarction (STEMI). We prospectively studied the diagnostic accuracy of two novel (T1, T2 mapping) and one established (T2 STIR) MRI methods for imaging the ischaemic area-at-risk (AAR) in patients with a recent NSTEMI.

## Methods

NSTEMI patients underwent contrast-enhanced cardiac MRI at 3.0 Tesla after percutaneous coronary intervention (PCI). The presence/extent of infarction was assessed with late gadolinium enhancement imaging (Gadovist, 0.1 mmol/kg). The infarct-related territory (IRA) was identified independently using a combination of angiographic, ECG and clinical findings. AAR was assessed with T1, T2 and T2 STIR methods by 2 observers who were blind to all of the clinical data. Comparisons were made between MRI and clinical findings.

## Results

Seventy-three NSTEMI patients (mean age 57 ± 10 yrs, 78% male) underwent 3TMRI. The mean infarct size was 5.5 ± 7.2% of left ventricular (LV) volume. The AAR T1 and T2 times (ms) were 1323 ± 68 ms and T2 57 ± 5 ms, respectively. The extent of AAR (% of LV volume) estimated with T1 (15.8 ± 10.6%) and T2 maps (16.0 ± 11.8%,) was similar (p = 0.838), and moderately well correlated (r = 0.82, P < 0.001). The 95% limits of agreement for mean area-at-risk estimated with T1 versus T2 maps were -13% and 13%. Mean AAR estimated with T2 STIR (7.8 ± 11.6%) was significantly lower than that estimated with T1 (P < 0.001) or T2 maps (P < 0.001). There were moderate correlations between AAR estimated with T1 maps vs. T2 STIR (r = 0.54, P < 0.001), and AAR estimated with T2 maps vs. T2 STIR (r = 0.46, P < 0.001). The 95% limits of agreement for mean myocardial AAR estimated with T1 vs. T2 STIR maps were -28% and 12% and for T2 vs. T2 STIR maps -32% and 16%. The IRA was correctly identified in 52 patients (71%) when using T1 maps, 56 (77%) for T2 maps, and 32 (44%) for T2 STIR maps. There was no difference in diagnostic accuracy with T1 and T2 maps (P = 0.125). A difference in diagnostic accuracy was observed between T1 maps and T2 STIR (P < 0.001), and T2 maps and T2 STIR (P < 0.001) for detecting IRA. Inter-observer agreement of infarct-related artery assignment was moderately high when analysed with T1 (κ = 0.790, P < 0.001) and T2 (κ = 0.794, P < 0.001) maps, but low with T2 STIR (κ = 0.555, P < 0.001).

## Conclusions

T1 and T2 maps have much higher diagnostic accuracy than T2 STIR maps, implying superior clinical utility.

## Funding

This study was funded through the British Heart Foundation and Health Science Scotland.

